# Characteristics, fates and complications of long-term silicone oil tamponade after pars plana vitrectomy

**DOI:** 10.1186/s12886-020-01608-5

**Published:** 2020-08-17

**Authors:** Nakhleh E. Abu-Yaghi, Yazan A. Abu Gharbieh, Ahmad M. Al-Amer, Saif Aldeen S. AlRyalat, Mohammed B. Nawaiseh, Mohammad J. Darweesh, Leen R. Alkukhun, Alaa M. Abed, Omar A. Saleh, Osama H. Ababneh

**Affiliations:** 1 Department of Special Surgery, Ophthalmology Division, the University of Jordan, P.O. Box 7599, Amman, 11118 Jordan; 2 School of Medicine; the University of Jordan, Amman, Jordan; 3 Department of Ophthalmology, Jordan University of Science and Technology, Ar-Ramtha, Jordan

**Keywords:** Silicone oil, Pars plana vitrectomy, Retinal detachment, Intraocular pressure, Ocular hypertension

## Abstract

**Background:**

Silicone oil tamponade has become a mainstay in treatment of advanced retinal detachment due to multiple etiologies. The aim of this study is to assess the characteristics, fates and complications of long-term silicone oil tamponade after par plana vitrectomy (PPV), and to compare the outcomes of different silicone oil viscosities used in a cohort of consecutive patients.

**Methods:**

This is a retrospective comparative case series of eyes undergoing vitrectomy with silicone oil tamponade for retinal detachment by a single surgeon using different oil viscosities that were followed for one year with the silicone oil in situ. Visual acuity (VA), intraocular pressure (IOP) and complications associated with the follow up period were analyzed and compared.

**Results:**

Eighty-five eyes of 85 patients were included in this study. Forty three patients had 1000 centistoke (cs) oil injected and 42 patients had 5000cs oil utilized. Demographic, cause of retinal detachment and preoperative ocular characteristics were similar in both groups. Long term complications in both groups included ocular hypertension (67.4% vs 66.7%), keratopathy due to silicone oil emulsification and migration to the anterior chamber (7.0% vs 11.9%), recurrent retinal detachment (4.7% vs 19%) and epiretinal membrane formation (7% vs 19%). In the 1000cs oil group, there was no significant difference between baseline IOP and any subsequent visit. There was a significant difference between baseline IOP and visits at day 1 (with IOP difference of 2.61 mmHg (±6.5)) (*p* = 0.028), 1 month (with IOP difference of 3.52 mmHg (±8.1)) (*p* = 0.026), 4 months (with IOP difference of 6.38 mmHg (±9.3)) (*p* = 0.005), and one year (with IOP difference of 4.24 mmHg (±11.1)) (*p* = 0.048), all higher in the post-operative period in the 5000cs oil group. Excluding the first post-operative day, no significant difference was found for VA between baseline visits and subsequent visits for either silicone oil groups.

**Conclusion:**

In this cohort of patients with long-term silicone oil tamponade after PPV to treat retinal detachment, IOP increased significantly in patients who received 5000cs silicone oil. There was no significant difference between other complication rates in patients receiving either oil viscosities. Long term silicone oil tamponade remains a viable option in certain cases, and a vigilant follow up for complications is necessary to limit any adverse effects and improve visual and surgical outcomes.

## Background

Ever since their introduction in 1962, silicone oils have increasingly been used in the treatment of advanced retinal detachment (RD) due to multiple etiologies. Silicone oils as tamponading agents were used to provide anatomical reattachment of the retina in cases of rhegmatogenous RDs, giant retinal tears, proliferative vitreoretinopathy, proliferative diabetic retinopathy and ocular trauma [1, 2]. Their use was also studied in cases of macular hole repair in patients with high myopia, RD due to choroidal coloboma involving the disc, and uveitis associated hypotony [3, 4]. In comparison to intraocular gases, silicone oils offered a prolonged tamponade without the need for storage, dilution or preparation making them more suitable in cases of recurrent detachments, with a lower incidence of postoperative hypotony [5].

Silicone oil of 1000 cSt (sc) viscosity was found to be superior to sulfur hexafluoride in the treatment of complex retinal detachment [6]. In vitro retinal detachment models have shown that increasing silicone oil viscosity increases resistance to volume displacement, a mechanism through which oil tamponades the retina against the retinal pigment epithelium. This may play an important role in preventing retinal detachment [7]. Furthermore, increasing the extensional viscosity of silicone oil was found to reduce its tendency for emulsification [8].

Although silicone oil has been successfully used by retina surgeons as a tamponading agent for decades, it is known to cause complications, including the development of cataracts in phakic eyes, band keratopathy in corneas with oil-endothelial touch, recurrent retinal detachments with variable rates, increased intraocular pressure (IOP), ocular hypotony, silicone oil emulsification, iritis, endophthalmitis, anterior dislocation and subretinal migration of the oil [9–16].

Removal of silicone oil from the eye is usually performed promptly to avoid or reverse these complications. Typically, silicone oil removal surgery is scheduled within 3–6 months of its placement. Nonetheless, in certain cases, removal of the oil itself may be associated with complications that may actually worsen the clinical outcome including retinal re-detachment and hypotony. In these usually complex and advanced cases, long or indefinite tamponade with silicone oil remains a viable option [17, 18].

Studies assessing the outcomes of long-term silicone oil tamponade and specifically comparing the extended outcomes of different viscosities of silicone oil are limited. Although no significant difference in the rates of complications between different oil viscosities has been reported, some studies have noted that oils with higher viscosities tend to emulsify later in vivo [2, 9].

We conducted this study to assess the characteristics, fates and complications of long-term silicone oil tamponade after pars plana vitrectomy (PPV) to treat retinal detachment, and to compare outcomes among patients retaining silicone oil of different viscosities.

## Methods

### Study design and data collection

We were given permission by the institutional review board at Jordan University Hospital (Approval # 10/2017/3749) to conduct this retrospective comparative review of patients who underwent PPV to treat rhegmatogenous or combined rhegmatogenous/tractional RD with silicone oil injection between 2010 and 2017. All patients provided written informed consent. For all participants, existing medical records were reviewed, and no patients’ identifiers were kept in record. We identified 43 consecutive patients who received 1000cs oil tamponade and compared them to 42 consecutive cases receiving 5000sc oil within the same time period. In both groups, patients with silicone oil tamponade for 1 y or more were included. Exclusion criteria included follow up of less than 1 year after surgery, incomplete or missing medical records, and cases with previous history of retinal detachment surgery in the same eye.

Data collected included demographic information, past ocular history, diagnosis/indication for surgery, Snellen best corrected visual acuity (BCVA), slit lamp examination details, IOP measured by Goldmann tonometry and fundus examination. Additional information documented included intra-operative and post-operative complications and duration of follow up after surgery.

Postoperative data retrieved included standard Snellen chart VA, slit lamp bio-microscopy examination details, fundus examination and IOP measurements by applanation with the Goldmann tonometer. Any information obtained by ancillary testing (including visual field testing and optical coherence tomography imaging) was also noted and documented. Data was collected on day 1, week 1, first month, 4 months and 12 months post-operatively. Other silicone oil complications as keratopathy, oil emulsification, retinal detachment, and optic neuropathy (as determined by clinical appearance of the disc and/or visual field or optical coherence tomography nerve fiber layer changes) were collected as present or not. Ocular hypertension was defined as IOP of > 21 mmHg.

### Surgical setting and technique

All surgeries were performed by one vitreo-retinal surgeon (NAY) with the same surgical setting for each case. We used a 20-gauge vitrectomy system (DORC International BV, Zuidland, Netherlands). Vitrectomy surgery included the standard 3-pars plana ports (1 port for infusion and 2 ports for intraocular instrumentation). After general anesthesia, the eye was prepared under sterile conditions including conjunctival disinfection with 5% povidone. All phakic patients underwent standard phacoemulsification and posterior chamber intraocular lens implantation. Starting with the infusion port, three pars plana sclerotomies were created using a 20-gauge MVR blade.

Vitrectomy parameters were set for proportional dual mode vitrectomy with cutting rate up to 2500 cpm and aspiration rate up to 200 mmHg. A panoramic view was used in all surgeries. Intravitreal triamcinolone acetonide was used in some cases to ease the visualization of vitreoretinal adhesion points if deemed necessary by the surgeon. Core vitrectomy was carried out and posterior hyaloid separation, if not already present, was performed by aspiration and mechanical lifting of the peripapillary cortical vitreous using the vitrectomy probe. Peripheral vitreous shaving with scleral depression was performed in all cases and end-gripping intraocular forceps were used to peel tractional membranes if present, without peeling the internal limiting membrane. Retinal breaks were identified and marked using endo-cautery and the retina was flattened using heavy perfluorocarbon liquid. Laser retinopexy around the breaks and 360 degrees was administered using a 20-gauge curved laser probe. At the end of the surgery depressed examination of the periphery was done under the microscope to check for peripheral breaks. Next, the heavy liquid was aspirated and complete fluid-air exchange was performed using the vitrector or the soft-tip cannula. Silicone oil was injected manually for a near-complete fill and the infusion cannula was removed. The choice of oil viscosity was subject to availability in the operating room. The sclerotomies were sutured with 8/0 vicryl and checked for silicone leak and the conjunctiva was also sutured. Topical antibiotic ointment was then applied, and the eye was covered with a patch and a shield.

### Statistical analysis

We used SPSS version 21.0 (Chicago, USA) in our analysis. We used mean (± standard deviation) to describe continuous variables (i.e. age, IOP, and VA). We used count (frequency) to describe other nominal variables (i.e. gender, laterality and others).

After testing for distribution via bar charts and sphericity via Mauchly’s test, we used paired sample t-test to study the mean difference between baseline IOP and IOP during subsequent visits, and we presented data in mean (95% confidence interval (CI)). Due to violation of normality assumption, we used Wilcoxon Signed Rank test to analyze the change in VA between baseline visit and subsequent visits, and we reported the results in number of entries that had a positive difference (improved visual acuity as compared to previous visits), negative difference (worsened visual acuity as compared to previous visits), and ties (no difference in visual acuity).

To analyze the difference in VA and IOP between patients with 1000sc and 5000sc silicone oil, we used independent samples T-test to assess the difference between IOP mean readings between the two silicone oil groups, where Levene’s test was used to evaluate equality of variances assumption. Due to violation of the normality assumption, we used Mann-Whitney U test to assess if there is a difference in VA readings between the 1000sc and 5000sc silicone oil groups. We adopted a *p* value of 0.05 as a significant threshold.

## Results

We included a total of 85 patients in this study with a mean age of 54.44 years (±15.3). Fifty-five (64.7%) patients were men and 30 (35.3%) were women. A total of 43 (50.6%) patients had 1000cs silicone oil implanted for one year, with a mean age of 58 (±14). They were 25 (58.1%) men and 18 (41.9%) women. A total of 42 (49.4%) patients had 5000cs silicone oil implanted for one year; with a mean age of 52 (±15). They were 30 (71.4%) men and 12 (28.6%) women. Detailed patients’ characteristics with the two oil viscosities are presented in Table 1.

**Table 1:** Characteristics of patients with 1000cs and 5000cs silicone oil

		**1000cs**			**5000cs**		
		**Mean ± SD**	**Count**	**Column N %**	**Mean ± SD**	**Count**	**Column N %**
**Age**		58 ± 14			51.5 ± 15		
**Sex**	Male		25	58.1%		30	71.4%
	Female		18	41.9%		12	28.6%
**Laterality**	Right		18	41.9%		28	68.3%
	Left		25	58.1%		13	31.7%
**History of Glaucoma**	Positive		5	11.6%		4	9.5%
**History of Ocular Surface Disease**	Positive		2	4.7%		6	14.3%
**Macula**	On		8	18.6%		6	14.3%
	Off		35	81.4%		36	85.7%
**Tractional membranes**	With		11	25.6%		14	33.3%
	Without		32	74.4%		28	66.7%
**Postoperative Keratopathy**	Positive		3	7.0%		5	11.9%
**Postoperative Ocular Hypertension**	Positive		29	67.4%		28	66.7%
**Recurrent RD**	Positive		2	4.7%		8	19.0%
**Postoperative ERM**	Positive		3	7.0%		8	19.0%
**VA Baseline**		.099 ± .161			0.107 ± .209		
**VA Day 1**		.056 ± .093			0.053 ± .063		
**VA Week 1**		.060 ± .086			0.062 ± .077		
**VA Month 1**		.070 ± .094			0.064 ± .067		
**VA Month 4**		.12845 ± .27397			0.089 ± .05766		
**VA Last visit**		.0597 ± .0622			0.056 ± .0638		
**IOP Baseline**		13.19 ± 2.59			11.83 ± 4.96		
**IOP Day 1**		11.73 ± 4.89			14.48 ± 5.94		
**IOP Week 1**		14.97 ± 8.22			13.86 ± 7.50		
**IOP Month 1**		16.68 ± 9.59			14.57 ± 6.82		
**IOP Month 4**		16.26 ± 8.85			16.07 ± 7.81		
**IOP Last visit**		16.17 ± 8.62			16.08 ± 10.70		

*SD* Standard Deviation, *RD* Retinal detachment, *ERM* epiretinal membrane, *VA* visual acuity, *IOP* intraocular pressure

For the 1000cs silicone oil group, we observed the following post-operative complications: 3 (7.0%) patients had keratopathy, 29 (67.4%) patients had ocular hypertension (defined as an IOP > 21 mmHg), 2 (4.7%) patients had recurrent RD, and 3 (7.0%) patients had an epiretinal membrane (ERM) formation. Regarding the post-operative complications for the 5000cs silicone oil group, 5 (11.9%) patients had keratopathy, 28 (66.7%) patients had ocular hypertension, 8 (19%) patients had recurrent RD, and 8 (19%) patients had an ERM.

Upon analyzing the IOP post-operatively compared to pre-operative baseline IOP for the 1000cs silicone oil group, we did not find a significant difference between baseline and any visit. The IOP peaked at 1 month with a mean difference between baseline IOP and IOP at 1 month post-operatively of 3.46 mmHg (±9.38) (*p* = 0.072). Table 2 details the comparison between baseline IOP and post-operative IOP at each visit for the 1000cs silicone oil group.

**Table 2:** Comparing baseline IOP with each visit for 1000cs and 5000cs silicone oil groups

	**Paired Differences**							
	**1000cs**				**5000cs**			
	**Mean ± SD**	**Std. Error Mean**	**95% CI of the Difference**	**Sig. (2-tailed)**	**Mean ± SD**	**Std. Error Mean**	**95% CI of the Difference**	**Sig. (2-tailed)**
**Pair 1 IOP Baseline – IOP Day 1**	1.94 ± 5.68	1.02	[−0.15, 4.02]	.068	−2.61 ± 6.52	1.14	[−4.91, −0.29]	.028
**Pair 2 IOP Baseline – IOP Week 1**	−1.74 ± 8.35	1.49	[−4.8, 1.32]	.255	−1.97 ± 8.9	1.59	[−5.2, 1.29]	.227
**Pair 3 IOP Baseline – IOP Month 1**	−3.46 ± 9.38	1.83	[−7.2, 0.32]	.072	−3.52 ± 8.07	1.49	[−6.58, −0.44]	.026
**Pair 4 IOP Baseline – IOP Month 4**	−1.73 ± 7.68	1.51	[−4.8, 1.37]	.262	−6.38 ± 9.28	2.02	[− 10.6, − 2.15]	.005
**Pair 5 IOP Baseline – IOP Last visit**	−2.71 ± 8.89	1.68	[− 6.1, − 0.73]	.118	− 4.24 ± 11.06	2.05	[−8.4, − 0.03]	.048

*SD* Standard Deviation, *CI* Confidence Interval, *IOP* Intraocular Pressure

On the other hand, upon analyzing the IOP post-operatively compared to the pre-operative baseline for the 5000cs silicone oil group, we found a significant difference between baseline IOP and visits on day 1 (with IOP difference of 2.61 mmHg (±6.5) higher in post-operative) (*p* = 0.028), 1 month (with IOP difference of 3.52 mmHg (±8.1) higher in post-operative)(*p* = 0.026), 4 months (with IOP difference of 6.38 mmHg (±9.3) higher in post-operative)(*p* = 0.005), and last visit (with IOP difference of 4.24 mmHg (±11.1) higher in post-operative)(*p* = 0.048).

All patients with increased IOP were managed with one or two topical anti-glaucoma medications. None of them required surgical intervention during the period of the study. Table 2 details the comparison between baseline IOP and post-operative IOP at each visit for the 5000cs silicone oil group.

For VA in the 1000cs silicone group, we found a significant difference in VA between baseline and first visit (*p* = 0.014), with 18 patients having a negative difference, 9 patients with positive difference, and 8 ties. For VA in the 5000cs silicone group, we only found a significant difference between the baseline visit and 1st day visit (*p* = 0.039), with 22 patients having a negative difference, 11 patients with positive difference, and 7 ties. Excluding the first post-operative day, no significant difference was found for VA between baseline visit and subsequent visits for either silicone groups.

Upon comparing the IOP between patients with 1000cs and 5000cs silicon oil groups, we only found a significant difference at day 1, where the 5000cs group had a mean IOP difference of 2.76 mmHg (95% CI: 0.15 to 5.37 mmHg) higher than 1000cs group (*p* = 0.039) (Table 3). No significant difference in VA between 1000cs and 5000cs silicon oil groups was noted (Table 4).

**Table 3:** Comparing mean IOP readings in each visit between 1000 and 5000 silicone oil groups

**Visit**	**Mean difference**	**t**	**df**	**Sig. (2-tailed)**	**95% CI of the difference**	**Effect size (Cohen’s d)**
**IOP Baseline**	1.36	1.46	71	.149	[−.497, 3.21]	0.342
**IOP Day 1**	−2.76	− 2.11	68	.039*	[−5.37, −.146]	0.507
**IOP Week 1**	1.10	.583	67	.562	[−2.67, 4.88]	0.14
**IOP Month 1**	2.10	.997	59	.323	[−2.11, 6.32]	0.252
**IOP Month 4**	0.18	.079	51	.937	[−4.42, 4.79]	0.021
**IOP Last visit**	0.078	.032	62	.975	[−4.82, 4.97]	0.008

*CI* confidence intervals, *IOP* Intraocular Pressure

* Significant at 0.05 level

**Table 4:** Comparing mean VA readings in each visit between 1000 and 5000 silicone oil groups

**Visit**	**Silicon Oil type**	**Range**	**Interquartile Range**	**Median**	**U**	**Sig. (2-tailed)**	**Effect size (Cohen’s d)**
**VA Baseline**	1000	0.60	0.03	0.05	770.50	.93	0.019
	5000	0.40	0.11	0.04			
**VA Day 1**	1000	0.50	0.05	0.02	681.50	.70	0.086
	5000	0.20	0.05	0.02			
**VA Week 1**	1000	0.50	0.03	0.05	647.00	.71	0.083
	5000	0.32	0.03	0.05			
**VA Month 1**	1000	0.50	0.07	0.05	609.50	.82	0.053
	5000	0.25	0.04	0.05			
**VA Month 4**	1000	0.30	0.08	0.05	346.50	.12	0.401
	5000	0.16	0.03	0.05			
**VA Last visit**	1000	0.30	0.08	0.02	530.50	.45	0.179
	5000	0.20	0.03	0.02			

*U* Mann-Whitney U test value

*VA* Visual Acuity

## Discussion

In this analysis we looked at 85 consecutive patients who underwent silicone oil tamponade with either 1000cs oil or 5000cs oil after PPV for RD. All those patients were followed for one year with the silicone in situ, and the long-term outcomes of both groups were observed and compared. Intraocular pressure was found to be significantly higher in the 5000sc oil group.

Despite some documented negative features, silicone oil remains the most compatible material for vitreous replacement [18]. Studies have not shown a difference in the tamponading force of various silicone oils, and selecting the proper oil for clinical use remains controversial [19–21]. Early postoperative rise in IOP is common after PPV with silicone oil injection and may be related to anterior chamber inflammatory activity or obstruction to aqueous flow by choroidal effusion or both [22]. The incidence of raised IOP varies from 3 to 40% and different studies reported varying prevalence rates of elevated IOP in patients receiving conventional silicone oil tamponade [22–24]. A study by Scott et al. comparing silicone oils of different viscosities used to treat retinal detachment concluded that anatomic and visual outcomes, as well as complication rates, were similar regardless of oil viscosity used [2].

Few studies have addressed the long-term effects of silicone oil tamponade after PPV [23]. Most surgeons advocate removal of silicone oil after 3–6 months, although some studies have showed that long term or indefinite tamponade is acceptable in certain cases [24]. A previous study done on 100 eyes with rhegmatogenous retinal detachment has shown the rate of keratopathy to be 6% [19], while in our study 8 out of the 85 patients developed postoperative keratopathy, representing 9.4% of the population, 3 of which were from the 1000cs silicone oil group (7% of that group) and 5 from the 5000cs silicone oil group (11.9%).

The rates of recurrent RDs in the literature ranged between 5 and 31.4% [20]. In our study, 10 patients had a recurrent RD, representing 11.8% of the population, 2 of which were from the 1000cs group (4.7% of the group) while 8 were from the 5000cs group (19% of the group). Furthermore, 11 out of the 85 patients developed ERM, representing 12.9% with 3 being from the 1000cs group (7% of the group) and 8 being from the 5000cs group (19%). The intraoperative use of intravitreal steroids with or without silicone oil to prevent postoperative proliferative vitreoretinopathy is still controversial, and no consensus exists for their use [25].

In a study conducted by by Ramezani et al. that included 263 eyes, 34 eyes (14.1%) developed ocular hypertension (IOP > 21 mmHg) [21]. Our study showed a much higher rate of this complication with 57 out of the 85 patients developing postoperative ocular hypertension, at 67.1% of the study subjects, 29 of which belonged to the 1000cs group (67% of the group), while 28 were from the 5000cs group (66% of the group). Rahman et al. compared IOP measurements after PPV with silicone oil placement due to RD in two groups who had different silicone oil viscosities (1000cs and 5000cs) placed. Their study concluded that although there was a difference between the IOP in both groups, it was not statistically significant [22] . In this study IOP was measured for all patients preoperatively as a baseline, and followed up postoperatively on the 1st day, 1st week, 1st month, 4th month and at one year and were compared to the baseline. In the 1000cs silicone oil group, intraocular pressure increased and peaked at 1 month with a mean difference of 3.46 mmHg (± 9.38) from the baseline, which was not statistically significant. On the other hand, in 5000cs silicone oil group, intraocular pressure significantly increased overtime, peaking at the 4th month with a mean difference of 6.38 mmHg (±9.3) (*p* = 0.005). No statistically significant difference has been found pertaining to other complication rates in both groups.

This study is limited by its retrospective nature and the number of cases analyzed. Patients in both groups were comparable in terms of various characteristics and pre-operative diagnosis, and those with incomplete information were not included. We have excluded eyes with previous detachments, and all analyzed cases where surgically handled by a single surgeon bringing confounders to a minimum.

## Conclusion

In this cohort of 85 patients with extended placement of silicone oil for the treatment of retinal detachment, long term complications in both groups of either silicone oil viscosities (1000cs and 5000cs) included ocular hypertension, keratopathy due to silicone oil emulsification and migration to the anterior chamber, recurrent retinal detachment and epiretinal membrane formation. There was no significant difference between the complication rates in patients with long-term silicone oil of either oil viscosity. Intraocular pressure increased significantly in patients who received silicone oil of the 5000cs viscosity. Long term tamponade remains a viable option in certain cases, and treating surgeons need to keep a watchful eye to detect and manage complications as they arise.

## Data Availability

Data sets used in this study are available from the contributing author upon reasonable request.

## Abbreviations

PPV: Pars Plana Vitrectomy
VA: Visual Acuity
IOP: Intraocular Pressure
Cs: Centistoke
RD: Retinal Detachment
